# Correction and Republication: Prevalence and Characteristics of Autism Spectrum Disorder Among Children Aged 8 Years — Autism and Developmental Disabilities Monitoring Network, 11 Sites, United States, 2012

**DOI:** 10.15585/mmwr.mm6745a7

**Published:** 2018-11-16

On April 1, 2016, *MMWR* Surveillance Summaries published “Prevalence and Characteristics of Autism Spectrum Disorder Among Children Aged 8 Years—Autism and Developmental Disabilities Monitoring Network, 11 Sites, United States, 2012” (*1*). On June 5, 2018, the authors informed *MMWR* about a number of inadvertent errors throughout the report that resulted from reporting of autism spectrum disorder cases among persons who did not live in the geographic surveillance area. Corrections of these errors do not change the interpretation or the conclusions of the original report. In accordance with December 2017 guidance from the International Committee of Medical Journal Editors (*2*), *MMWR* has corrected and republished the report (*3*). The republished report includes the original report with clearly marked corrections in supplementary materials.
